# Fast-Strain Encoded Cardiac Magnetic Resonance During Vasodilator Perfusion Stress Testing

**DOI:** 10.3389/fcvm.2021.765961

**Published:** 2021-11-17

**Authors:** Henning Steen, Moritz Montenbruck, Sebastian Kelle, Sebastian Esch, Arne Kristian Schwarz, Sorin Giusca, Grigorios Korosoglou

**Affiliations:** ^1^Medneo, Hamburg, Germany; ^2^Cardiology/Cardiac Imaging, Marien Hospital, Hamburg, Germany; ^3^Department of Internal Medicine/Cardiology, Deutsches Herzzentrum Berlin, Berlin, Germany; ^4^Department of Internal Medicine and Cardiology, Charité—Universitätsmedizin Berlin, Campus Virchow-Klinikum, Berlin, Germany; ^5^Deutsches Zentrum für Herz-Kreislauf-Forschung (DZHK) (German Centre for Cardiovascular Research), Berlin, Germany; ^6^Department of Cardiology, Vascular Medicine and Pneumology, Gesundheitszentren Rhein-Neckar (GRN) Hospital Weinheim, Weinheim, Germany; ^7^Cardiac Imaging Center Weinheim, Hector Foundation, Weinheim, Germany

**Keywords:** fast strain-encoded CMR (fast-SENC), average perfusion score index, adenosine, late gadolinium enhancement, cardiac outcomes

## Abstract

**Background:** Cardiac magnetic resonance perfusion imaging during vasodilator stress is an established modality in patients with suspected and known coronary artery disease (CAD).

**Aim:** This study aimed to evaluate the performance of fast-Strain-Encoded-MRI (fast-SENC) for the diagnostic classification and risk stratification of patients with ischemic heart disease.

**Methods:** Perfusion and fast-SENC cardiac magnetic resonance (CMR) images were retrospectively analyzed in 111 patients who underwent stress CMR. The average myocardial perfusion score index, global and segmental longitudinal and circumferential strain (GLS and GCS and SLS and SCS, respectively), were measured at rest and during stress. The combination of SLS and SCS was referred to as segmental aggregate strain (SAS). Segments exhibiting perfusion defects or SAS impairment during stress were defined as “ischemic.” All-cause mortality, non-fatal infarction, and urgent revascularization were deemed as our combined clinical endpoint.

**Results:** During adenosine stress testing, 44 of 111 (39.6%) patients exhibited inducible perfusion abnormalities. During a mean follow-up of 1.94 ± 0.65 years, 25 (22.5%) patients reached the combined endpoint (death in *n* = 2, infarction in *n* = 3 and urgent revascularization in *n* = 20). Inducible perfusion defects were associated with higher number of segments with inducible SAS reduction ≥6.5% (χ^2^ = 37.8, AUC = 0.79, 95% CI = 0.71–0.87, *p* < 0.001). In addition, patients with inducible perfusion defects or SAS impairment exhibited poorer outcomes (AUC_Perf_ = 0.81 vs. AUC_SAS_ = 0.74, *p* = NS vs. each other, and χ^2^ = 30.8, HR = 10.3 and χ^2^ = 9.5, HR = 3.5, respectively, *p* < 0.01 for both).

**Conclusion:** Purely quantitative strain analysis by fast-SENC during vasodilator stress was related to the diagnosis of ischemia by first-pass perfusion and is non-inferior for the risk stratification of patients with ischemic heart disease. This may bear clinical implications, especially in patients with contraindications for contrast agent administration.

## Introduction

Cardiovascular diseases are the main cause of morbidity and death in the world, annually claiming more lives than all forms of cancer together (1). Non-invasive anatomical imaging of coronary arteries by cardiac computed tomography and functional stress testing like cardiac magnetic resonance (CMR) is currently recommended as first-line diagnostic techniques in patients with suspected or known coronary artery disease (CAD) (2, 3). In this regard, stress CMR is a well-established method for the diagnostic classification and risk stratification of such patients (4–8). Especially vasodilator stress CMR is widely used due to its excellent safety profile and superior accuracy compared with scintigraphy (5, 8, 9).

Currently, the detection of inducible ischemia during vasodilator stress CMR is mostly based on the visual assessment of perfusion defects (5–8), which is subjective and depends on the experience of the readers. In this regard, fast-Strain-Encoded-MRI (fast-SENC) has been utilized for the objective assessment of longitudinal and circumferential myocardial strain (LS and CS) in previous studies [summarized in (10)]. The ability of this technique to diagnose relevant CAD during inotropic stress and predict future adverse events has been previously demonstrated (11, 12). However, limited data are available on the value of fast-SENC to accurately identify perfusion defects and to provide risk stratification in patients who undergo vasodilator stress CMR.

Therefore, we sought to determine the diagnostic and prognostic value of SENC during vasodilator stress in patients with known or suspected CAD. Global LS (GLS) and CS (GCS) and average global strain (GS), as well as segmental LS (SLS) and CS (SCS) and combined information from SLS or SCS, referred to as segmental aggregate strain (SAS) were compared with perfusion data.

## Methods

### Study Population

Our patient cohort consisted of patients who underwent adenosine stress CMR between September 2017 and July 2019 in the Marien Hospital Hamburg, Hamburg, Germany and had verified follow-up outcomes. Patients were referred for stress CMR due to known or suspected CAD based on current guidelines (2). CMR was performed as part of standard institutional protocols. All patients were above 18 years old and gave written informed consent. The study was conducted in concordance with the Declaration of Helsinki. Patients were excluded from the study in case of claustrophobia, contraindications to adenosine, such as the history of asthma, low blood pressure, clinical instability, advanced atrioventricular block, or sinus bradycardia, known allergy to gadolinium-based contrast agents, renal failure with a glomerular filtration rate (GFR) < 30 ml/kg/min or inability to give informed consent. All patients were deferred from consuming caffeine-containing beverages and food for 24 h before the examination. If this was not the case the examination was postponed to a later timepoint. Prior to the examination, demographic data, including the presence of arterial hypertension, hyperlipidemia, diabetes mellitus, and previous CAD were acquired.

### Cardiac Magnetic Resonance Examination

Examinations were performed using a 1.5 T MR system (Achieva, Philips Healthcare, Best, The Netherlands) equipped with cardiac phased-array receiver coils. Data acquisition was triggered on the R-wave using a 4-lead vector ECG. Cine images were obtained using a breath-hold segmented-k-space balanced fast-field echo sequence (SSFP), employing retrospective ECG gating in long-axis planes (2-, 4-, and 3-chamber views) and contiguous short-axis slices covering the whole ventricles, with typically reconstructed 30 phases per cardiac cycle with 50 and 67% acquired heart phases, respectively, for short and long-axis images.

### Adenosine Stress CMR Protocol and Image Acquisitions

A standard protocol for adenosine stress CMR was used, which is described in detail elsewhere (13). In short, patients received a continuous infusion of adenosine. The dose was varied between 140 and 210 μg/kg/min depending on heart rate change and clinical symptoms. During the infusion, patients were continuously monitored for heart rate and oxygen saturation, and blood pressure was measured every minute. The stress perfusion acquisition was started after at least 3–4 min of the adenosine infusion and when two criteria were met: increase in heart rate of at least 10–15 beats per minute and/or the blood pressure fall of at least 10 mmHg or both and the occurrence of symptoms related to the adenosine infusion. In case of no heart rate or blood pressure response, the dose of the adenosine infusion was increased to 175 or 210 μg/kg body weight/min until a response was observed. In addition, the presence of splenic switch-off was verified according to current recommendations (14). Stress acquisitions were performed using a gadolinium-based contrast agent (Dotarem^®^- gadoterate meglumine in a dosage of 0.05 mmol/kg). Three short-axis slices were acquired using a single-shot saturation recovery gradient echo sequence (FOV 320 mm, slice thickness = 8 mm, TE = 1.02 ms, TR = 226 ms, TI = 140 ms, Flip angle = 50°). Fast-SENC acquisitions were performed at baseline, and repeated acquisitions were performed in identical planes during infusion of adenosine after meeting the criteria mentioned above and before the administration of gadolinium for the acquisitions of first-pass perfusion scans. The rest perfusion was performed after 10 min using identical planes and the same dosage of the contrast agent. Afterward, late gadolinium enhancement (LGE) acquisitions were performed in three long-axis and multiple short axes, covering the entire left ventricle.

### Evaluation of Myocardial Perfusion

All analyses were performed on a commercially available workstation (CVI 42, Circle Cardiovascular Imaging Inc., Calgary, Canada). Results for ventricular volumes, left- and right-ventricular (LV and RV) ejection fraction (%), and myocardial mass were derived from short-axis slices. The presence of myocardial perfusion defects was performed visually in three short-axis images. A perfusion defect was defined as a region in the myocardium exhibiting hypo-enhancement by visual criteria that persists after peak myocardium enhancement for at least 4 RR intervals and corresponds to a coronary territory (15). In addition, semiquantitative perfusion analysis was performed by using a 3-point grading scale (16):

1 = Normal Perfusion,

2 = Perfusion Deficits With <50% Transmurality and

3 = Perfusion Deficits With ≥50% Transmurality During Adenosine Stress

Corresponding images in a patient with normal perfusion during stress, with a subendocardial and with a transmural perfusion defect during vasodilator stress are provided in our Supplementary Figure 1. Based on this grading system, an average perfusion score index was built for analysis by patients, by calculating the mean score in 17 myocardial segments, as recommended by the AHA (17). Images were analyzed by experienced operators with more than 10 years of experience in cardiovascular imaging and acquired level 3 certification by the German Society of Cardiology (HS & MM).

### Single Heartbeat Fast-SENC Acquisitions

As described previously, fast-SENC is based on the acquisition of high- and low-tuning image sequences with different frequency modulation. Fast-SENC image sequences were analyzed using the MyoStrain software (Myocardial Solutions, Inc., Morrisville, North Carolina, USA), as described previously (18).

With fast-SENC, bright regions in the two frequency modulation images represented static and fully contracted tissues, respectively. Circumferential and longitudinal strain within a range from 5 to −30% were encoded, with negative values translating into active myocardial contraction. In our study, a single heartbeat, a fast-SENC variant with single-shot spiral readouts was employed. Typical imaging parameters were as follows: field-of-view = 256 × 256 mm, slice thickness = 10 mm, voxel size = 4 × 4 × 10 mm, reconstructed resolution = 1 × 1 × 10 mm, single-shot spiral readout with acquisition time TA = 10 ms, flip angle = 30°, effective echo time (TE) = 0.7 ms, repetition time (TR) = 12 ms, temporal resolution = 36 ms, the typical number of acquired heart phases = 22, spectrally selective fat suppression (SPIR), and total acquisition time per slice < 1 s. Data were acquired in three long-axis (four-, three-, and two-chamber) views, and three short-axis views of the LV (basal, mid-ventricular, and apical).

Global circumferential strain by fast-SENC is extracted from 3 long-axis views, whereas GLS is extracted from the 3 short-axis images. The endocardial and epicardial borders were drawn at the end-systolic cardiac phase and are traced throughout the cardiac cycle, using an automatic tissue tracking algorithm. Tracking is then verified and manually corrected if necessary. A 16-segment model was used for the GLS and a 21-segment model for the GCS. For the analysis, GLS and GCS were expressed as the average value of all 16 and 21 segments, respectively. In addition, a GS was calculated by averaging LS and CS in all available segments (19, 20).

Global circumferential strain, GLS, and GS were measured both at baseline and during adenosine stress. In addition, segments with relevant segmental LS and/or CS strain (SAS) impairment during stress were defined as ischemic. The number of such “ischemic” segments was calculated for SLS, SCS, and SLS or SCS which was referred to as SAS in each patient.

Because myocardial shortening occurred in both longitudinal and circumferential directions during systole, the strain values were consequently negative and were so reported. However, throughout the text, and in keeping with most of the literature on the subject, we refer to the absolute values, i.e., higher strain values meaning more deformation and consequently more “negative” values.

### Definition of Study Endpoints

Personnel unaware of the CMR results contacted all patients or relatives of patients who underwent stress CMR studies during follow-up. All-cause mortality, the occurrence of non-fatal myocardial infarction, and urgent coronary revascularization by PCI or CABG were selected as the combined primary endpoint of our study. We used all-cause and not only cardiovascular mortality as an endpoint since the prior is free of potential subjectivity, clinically compelling, and therefore most relevant to the patients (21). In addition, urgent revascularization was fulfilled only if patients were hospitalized unexpectedly because of persisting or increasing chest pain and revascularization was performed urgently within the same hospitalization (22).

### Statistical Analysis

Data were presented as *M* ± *SD* for continuous variables and as absolute values and percentages for categorical values. A paired *t*-test was used to compare two groups of normally distributed values. The ANOVA test was used for comparing three or more normally distributed groups with the Scheffé test for *post-hoc* analysis (23). The Mann-Whitney test was used to compare ordinal variables and the Fisher test to compare nominal variables. A Pearson correlation test was employed to test the relation between strain and perfusion variables. A receiver operator characteristics (ROC) analysis was used to identify the best parameter that identifies the presence of perfusion abnormalities or cardiac endpoints. Comparison of the areas under the curve (AUC) of paired data ROC curves was performed using the DeLong method (24). Survival curves were estimated by the Kaplan-Meier method and compared by log-rank tests. In addition, a hierarchic logistic regression model was used to assess the incremental value of myocardial perfusion and strain to clinical variables (age, diabetes mellitus, and history of CAD) for the prediction of the combined endpoint by calculating the corresponding total χ^2^ values. Inter- and intra-observer variabilities for strain values were assessed by repeated analysis of 40 randomly selected patients and were calculated as the ratio of the standard deviation to the mean. Based on a statistical power of 90%, a two-sided type I error of 0.05, and the expected *SD* of the results, as well as the expected margins of error (pre-specified non-inferiority margin), we calculated that a minimum of 70 patients would be necessary for our study for a comparison between strain and conventional first-pass perfusion imaging. The MedCalc software version 20.009 (MedCalc, Ostend, Belgium, 2019) was used throughout. All *p* < 0.05 were considered statistically significant.

## Results

### Demographic, CMR, and Outcome Data

Complete CMR and follow-up data were available in 111 individuals who underwent vasodilator stress perfusion CMR for clinical reasons. Demographic, clinical, and CMR data are provided in Table 1. Mean age was 62.6 ± 11.8 years old whereas 28 (25%), 84 (76%), and 16 (14%) patients had diabetes mellitus, hyperlipidemia, and previous infarction, respectively.

**Table 1:** Demographic, clinical, and CMR data from our patient cohort.

	**All patients (*n* = 111)**	**Patients w/o inducible perfusion defects (*n* = 67)**	**Patients with inducible perfusion defects (*n* = 44)**	***P*** **-values**	**Patients w/o cardiac endpoints (Death/MI/PCI) (*n* = 86)**	**Patients with cardiac endpoints (Death/MI/PCI) (*n* = 25)**	***P*** **-values**
**Demographic data**							
Age (years)	62.6 ± 11.8	61.4 ± 12.5	64.4 ± 10.4	0.19	62.2 ± 12.6	63.8 ± 8.7	0.56
Male gender	76 (68%)	47 (70%)	29 (66%)	0.64	62 (72%)	14 (56%)	0.13
Arterial hypertension	88 (79%)	53 (79%)	35 (80%)	0.96	69 (80%)	19 (76%)	0.65
Type 2 diabetes mellitus	28 (25%)	20 (30%)	8 (18%)	0.17	20 (23%)	8 (32%)	0.38
Hyperlipidemia	84 (76%)	51 (76%)	33 (75%)	0.89	63 (73%)	21 (84%)	0.28
Past myocardial infarction	16 (14%)	10 (15%)	6 (14%)	0.85	11 (13%)	5 (20%)	0.37
Known CAD	74 (67%)	41 (61%)	33 (75%)	0.13	54 (63%)	20 (80%)	0.11
Body-mass-index (kg/m^2^)	27.0 ± 3.8	26.7 ± 4.0	27.4 ± 3.5	0.34	26.9 ± 4.0	27.2 ± 3.3	0.73
**Baseline CMR data**							
LV ejection fraction (%)	57.1 ± 7.2	57.1 ± 6.8	57.0 ± 7.9	0.96	56.7 ± 7.5	58.6 ± 6.1	0.25
IVS (mm)	11.0 ± 2.1	10.9 ± 2.1	11.2 ± 2.1	0.37	11.2 ± 2.1	10.4 ± 1.7	0.12
Lateral wall (mm)	7.3 ± 2.0	7.3 ± 1.8	7.3 ± 2.3	0.94	7.3 ± 2.0	7.4 ± 2.0	0.74
LV mass (g)	109.2 ± 25.2	104.7 ± 22.0	116.0 ± 28.4	0.02	109.2 ± 24.7	109.0 ± 27.6	0.97
LV mass index (g/m^2^)	56.2 ± 10.9	54.4 ± 8.9	59.0 ± 13.1	0.03	56.1 ± 11.0	56.6 ± 11.0	0.82
Native T1 values	1,042 ± 3,446	1,047 ± 32	1,037 ± 35	0.22	1,044 ± 32	1,037 ± 41	0.53
RV ejection fraction (%)	55.8 ± 6.2	56.5 ± 6.4	54.7 ± 5.6	0.12	55.8 ± 6.4	55.9 ± 5.5	0.98
Wall motion score index	1.16 ± 0.33	1.19 ± 0.38	1.11 ± 0.25	0.19	1.19 ± 0.37	1.05 ± 0.14	0.07
CAD related LGE score	1.13 ± 0.28	1.12 ± 0.30	1.13 ± 0.22	0.93	1.13 ± 0.30	1.12 ± 0.19	0.91
**Perfusion and strain CMR data**							
Perfusion defect (yes/no)	44 (40%)	67 (0%)	44 (100%)	N.A.	22 (26%)	22 (88%)	<0.001
Average perfusion score index	1.12 ± 0.19	1.0 ± 0.0	1.3 ± 0.18	<0.001	1.09 ± 0.15	1.28 ± 0.22	<0.001
GS (%) at baseline	−18.7 ± 2.0	−18.9 ± 1.96	−18.4 ± 2.0	0.18	−18.7 ± 1.9	−18.5 ± 2.1	0.57
GS (%) during stress	−19.4 ± 3.3	−19.8 ± 1.7	−18.9 ± 1.9	0.005	−19.5 ± 1.9	−19.2 ± 1.5	0.54

*CAD, coronary artery disease; IVS, intraventricular septum; LGE, late gadolinium enhancement; MI, myocardial infarction; PCI, percutaneous coronary intervention; LV, left ventricular; RV, right ventricular; GS, global strain; w/o, without; CMR, cardiac magnetic resonance*.

During the adenosine stress test, 44 of 111 (39.6%) patients exhibited inducible perfusion abnormalities. Patients with inducible perfusion abnormalities had similar demographic and baseline CMR data to those with negative stress results, except for LV-mass, which was increased in patients with inducible perfusion abnormalities. However, significant differences were observed with the average perfusion score index and for global strain parameters during stress in patients with vs. without perfusion abnormalities (Table 1).

### Association Between Myocardial Strain and Perfusion

**Figure 1:**
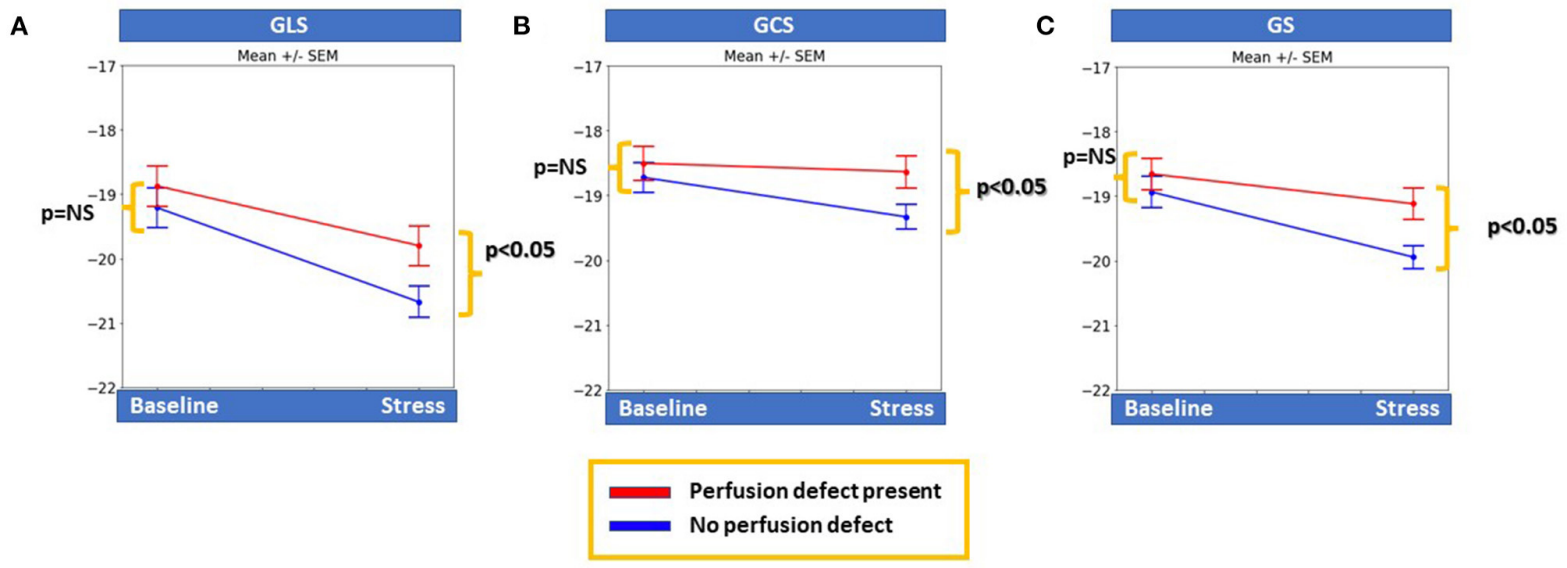
Patients with inducible perfusion abnormalities had significantly lower global longitudinal and circumferential myocardial strain (GLS and GCS, respectively), as well as global strain (GS) during stress compared to patients with negative stress results (*p* < 0.05 for all), whereas baseline strain values were similar for all markers between the two groups **(A–C)**.

Patients with inducible perfusion defects had significantly lower GLS, GCS, and GS during stress (*p* < 0.05 for all) compared with patients without perfusion defects, whereas all baseline strain values were similar between the 2 groups (Figures 1A–C).

**Figure 2:**
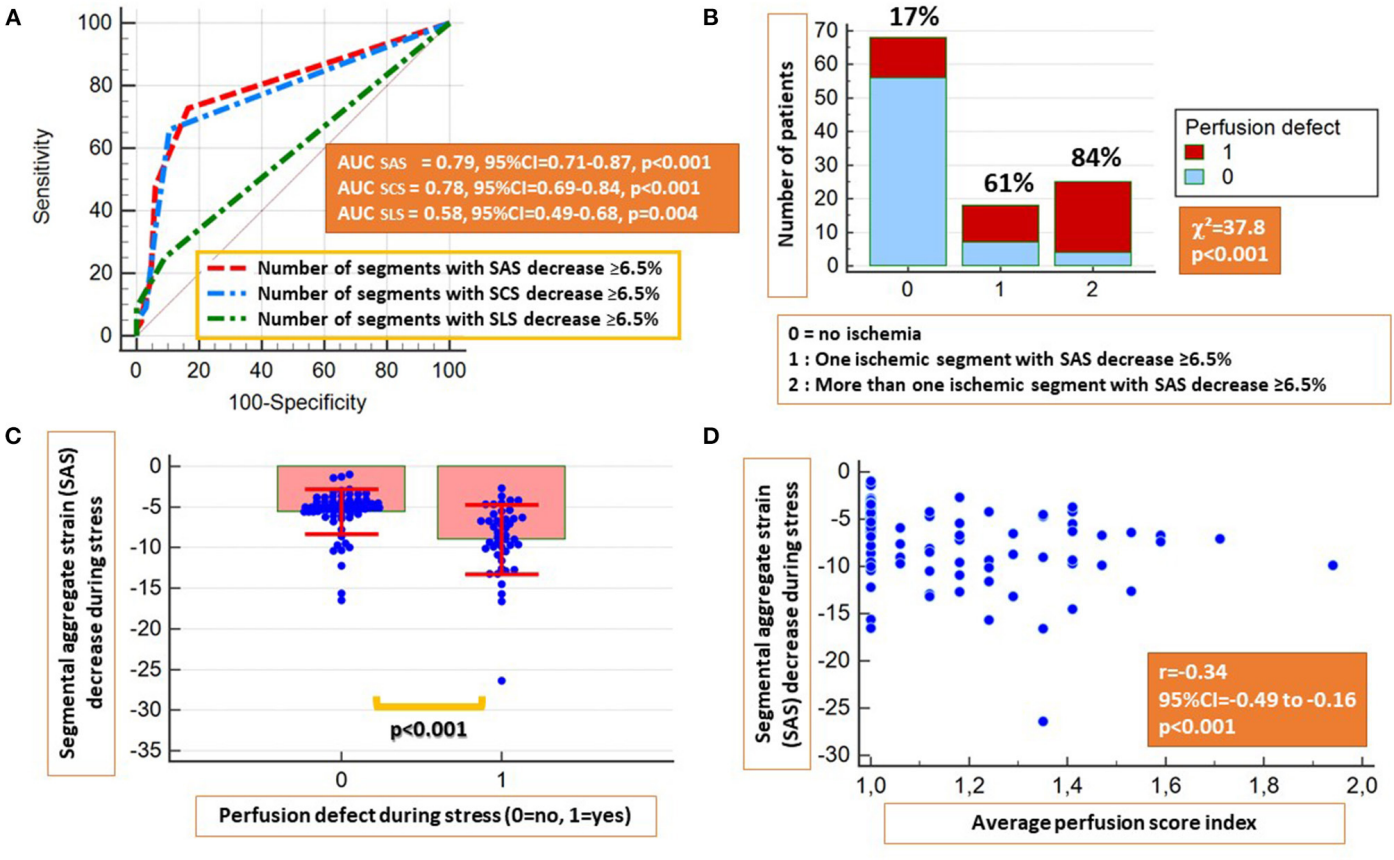
The presence of inducible strain decrease ≥6.5% by segmental LS (SLS), segmental CS (SCS), and segmental aggregate strain (SAS) during stress were all indicative for the presence of inducible perfusion abnormalities, with SCS and SAS exhibiting higher accuracies **(A)**. Patients with inducible perfusion abnormalities exhibited a higher number of ischemic segments by SAS **(B)** and higher absolute SAS decrease during stress **(C)**. A moderate inverse correlation was observed between the perfusion score index and inducible SAS decrease **(D)**.

Based on *a priori* ROC analysis, a cut-off value of 6.5% for absolute segmental strain (SLS, SCS, and SAS) reduction during vasodilator stress was selected as best indicative for the presence of segmental myocardial ischemia by perfusion analysis. Thus, the number of ischemic segments with inducible SLS, SCS, or SAS decrease ≥6.5% were all indicative for the presence of inducible perfusion defects, with SAS exhibiting the highest accuracy, followed by SCS and SLS (AUC_SCS_ = 0.78, 95% CI = 0.69–0.84, AUC_SAS_ = 0.79, 95% CI = 0.71–0.87 and AUC_SLS_ = 0.58, 95% CI = 0.49–0.68; *p* < 0.05 for SLS vs. SAS and SCS, Figure 2A). Therefore, SAS values deriving information both from longitudinal and circumferential deformation were used for further analysis. Corresponding sensitivities and specificities are provided in Table 2A.

**Table 2:** Sensitivities, specificities, and accuracy values for **(A)** detection of inducible perfusion abnormalities by strain and **(B)** Prediction of outcomes using perfusion and strain parameters.

	**Parameters**	**Criterion**	**Sensitivity**	**Specificity**	**AUC**	***p*** **-values**
**A. Presence of inducible perfusion abnormalities**						
Presence of perfusion defects	GS during stress (%)	>−19.8%	71%	64%	0.65	0.004
	SAS strain reduction ≥6.5%	≥6.5%	77%	81%	0.78	0.05 vs. SLS and *p* = NS vs. SCS
	Number of segments with SAS reduction≥6.5%	≥1 segment	73%	84%	0.79	
**B. Cardiac outcomes during follow-up**						
Prediction of cardiac endpoint during follow-up	Average perfusion score index	>1.06	82%	77%	0.82	P = NS for perfusion abnormalities or index vs. SAS
	Perfusion abnormality present	≥1	86%	76%	0.81	
	Number of segments with SAS reduction ≥6.5%	≥1	75%	74%	0.74	
	At least one segment with SAS reduction ≥6.5%	≥1	75%	74%	0.74	

*AUC, area under the curve; GS, global strain; SAS, segmental aggregate strain; SLS, segmental longitudinal strain; SCS, segmental circumferential strain*.

Patients with inducible perfusion defects exhibited a higher number of segments with inducible SAS decrease ≥6.5% (χ^2^ = 37.8, *p* < 0.001) and significantly higher absolute SAS decrease during adenosine stress (*p* < 0.001) (Figures 2B,C). In addition, a weak inverse correlation was observed between the average perfusion score and the SAS decrease during stress (*r* = −0.34, *p* < 0.01; Figure 2D). On a segmental level, a slight absolute SLS decrease and a blunted SLS increase were noticed in segments with subendocardial and transmural perfusion defects, respectively, compared with segments with normal perfusion during stress, exhibiting a slight SLS increase (Supplementary Figure 2A). The same patterns were observed after the exclusion of segments with LGE (Supplementary Figure 2B). Additional correlations between perfusion and strain parameters are provided in Table 3.

**Table 3:** Correlations between the average perfusion score index and strain parameters during stress.

**Parameters**	**GS during stress**	**Minimum SAS difference during stress**	**Number of segments with inducible SAS decrease ≥6.5%**
Average perfusion score	−0.20 *P* = 0.03	−0.34 *P* < 0.001	0.35 *P* < 0.001

*GS, global strain; SAS, segmental aggregate strain*.

### Association With Perfusion and SAS With Clinical Endpoints

During a mean follow-up duration of 1.94 ± 0.65 years, 2 patients died, 3 had a non-fatal myocardial infarction and 20 underwent urgent coronary revascularization by PCI (*n* = 15) or CABG (*n* = 5).

**Figure 3:**
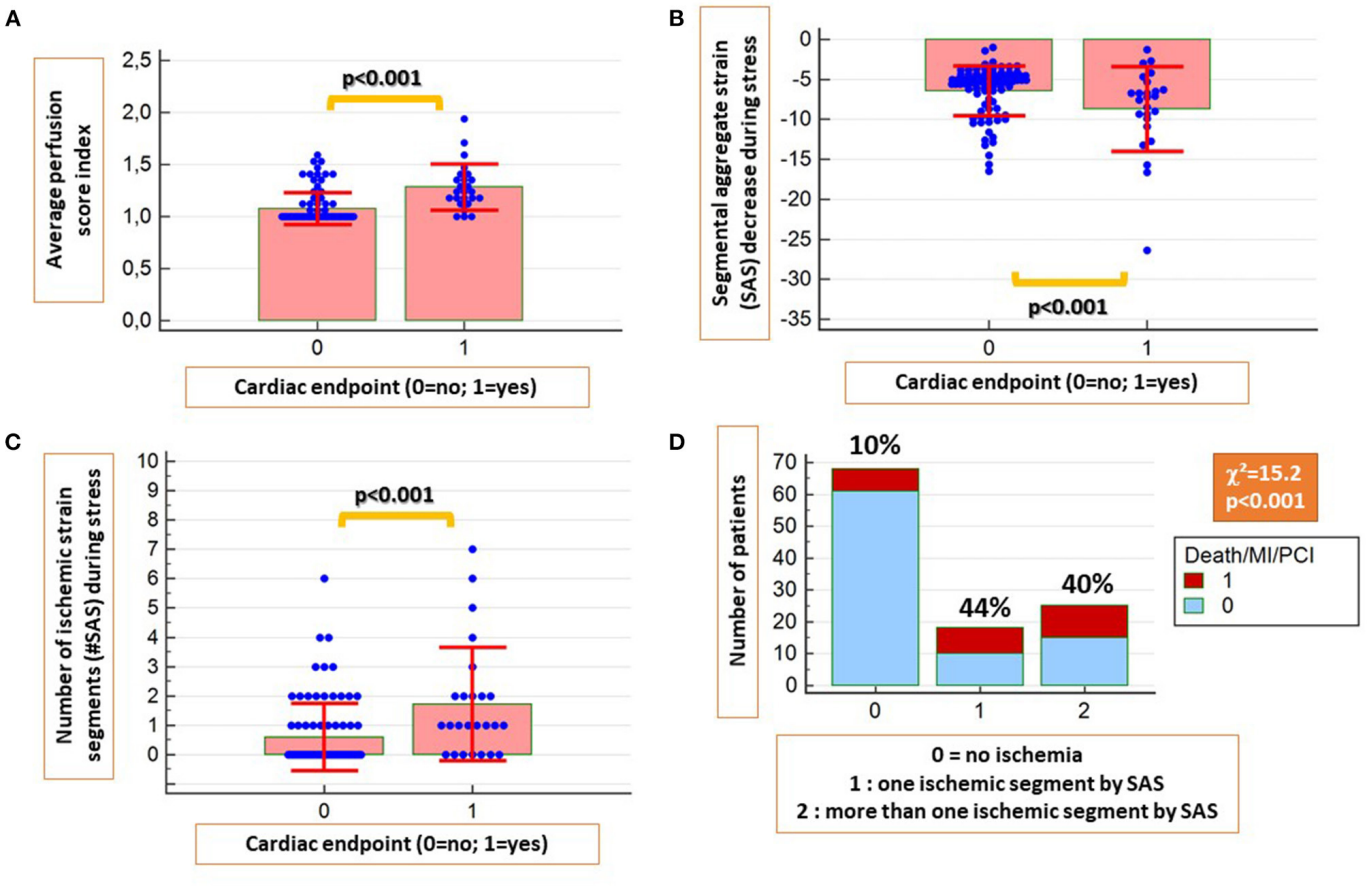
The average perfusion score index was significantly higher in patients with future cardiac endpoints **(A)**. Similarly, patients with cardiac endpoints had significantly higher absolute SAS decrease during stress **(B)** and a higher number of ischemic segments by SAS **(C)**. In addition, a strong association was present between ischemic segments by SAS and future cardiac events **(D)**.

A significant association was observed between inducible perfusion abnormalities and the combined endpoint (χ^2^ = 31.7, contingency coefficient = 0.47, *p* < 0.001). The perfusion score index was significantly higher in patients with vs. without cardiac endpoints (Figure 3A).

In addition, patients with cardiac endpoints exhibited higher SAS decrease and higher number of ischemic segments by SAS during stress compared with those without cardiac endpoints (*p* < 0.001 for both; Figures 3B,C). A strong association also was present between the presence of ischemic segments by SAS and future events (χ^2^ = 15.2, *p* < 0.001; Figure 3D).

### Prediction of Endpoints by Perfusion and Segmental Strain

**Figure 4:**
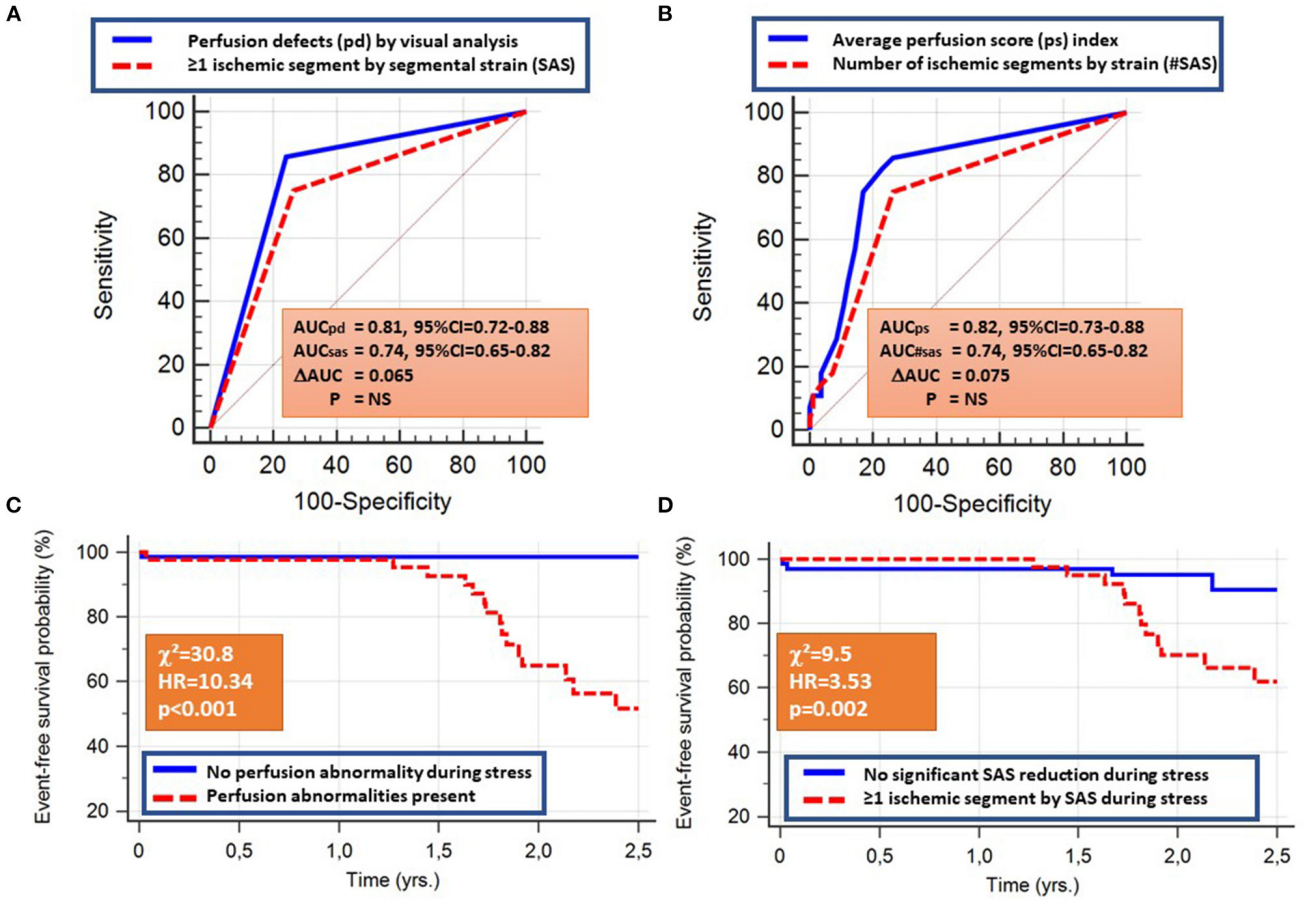
The presence of perfusion abnormalities or at least one ischemic segment by SAS, the average perfusion score, and the number of ischemic segments by SAS all predicted the combined cardiac endpoint with similar accuracy rates **(A,B)**. The corresponding Kaplan-Maier curves are provided in **(C,D)**.

Both visual perfusion and quantitative SAS analysis predicted the combined cardiac endpoint with similar accuracy rates (AUC = 0.81, 95% CI = 0.72–0.88 vs. 0.74, 95% CI = 0.65–0.82; ΔAUC =0.065, *p* = NS). Similarly, the perfusion score index performed as well as the number of ischemic segments by SAS, predicting the combined endpoint (AUC = 0.82, 95% CI = 0.73–0.88 vs.0.74, 95% CI = 0.65–0.82; ΔAUC = 0.075, *p* = NS; Figures 4A,B). The corresponding sensitivity and specificity values and Kaplan-Maier curves are provided in Table 2B and Figures 4C,D, respectively.

**Figure 5:**
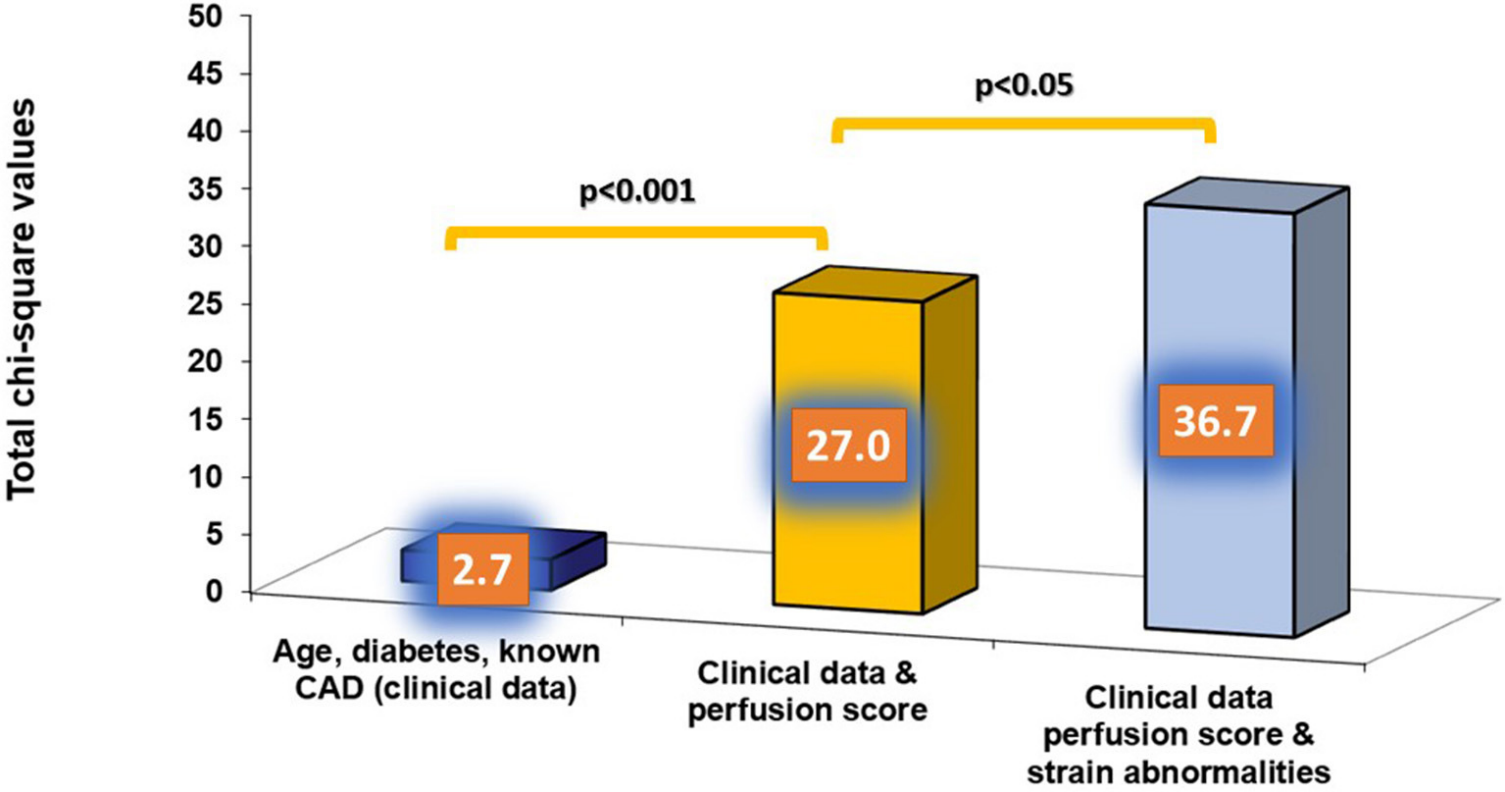
The presence of strain abnormalities added incremental value to perfusion imaging and clinical data for the prediction of the combined endpoint.

The independent association of perfusion and SAS with cardiac outcomes were confirmed by multivariable regression analysis (Table 4). In addition, the presence of impaired SAS during stress exhibited incremental value to perfusion imaging and clinical data for the prediction of the combined endpoint, as shown by the acquired χ^2^ values (Figure 5).

**Table 4:** Multiple regression analysis for the prediction of the combined endpoint death, non-fatal myocardial infarction, and urgent coronary revascularization during follow-up.

	**Coefficients**	**r_**partial**_**	***p*** **-values**
Age (yrs)	0.00035	0.01	0.91
LVEF (%)	0.0046	0.08	0.40
CAD related LGE score	−0.02	−0.013	0.88
Average perfusion score	0.82	0.36	0.0002
Ischemic segments	0.23	0.26	0.006
by SAS			

*LVEF, left ventricular ejection fraction; SAS, segmental aggregate strain; LGE, late gadolinium enhancement; CAD, coronary artery disease*.

### Intra and Interobserver Variabilities

Intra- and interobserver variabilities for LS, CS were 1.5 and 1.6% and 2.1 and 2.3%, respectively. Total acquisition time was <15 s per patient, whereas the time spent required for quantitative analysis of LS, CS, and SAS was 304 ± 125 s (5.1 ± 2.1 min) per patient.

### Patient Case Example

**Figure 6:**
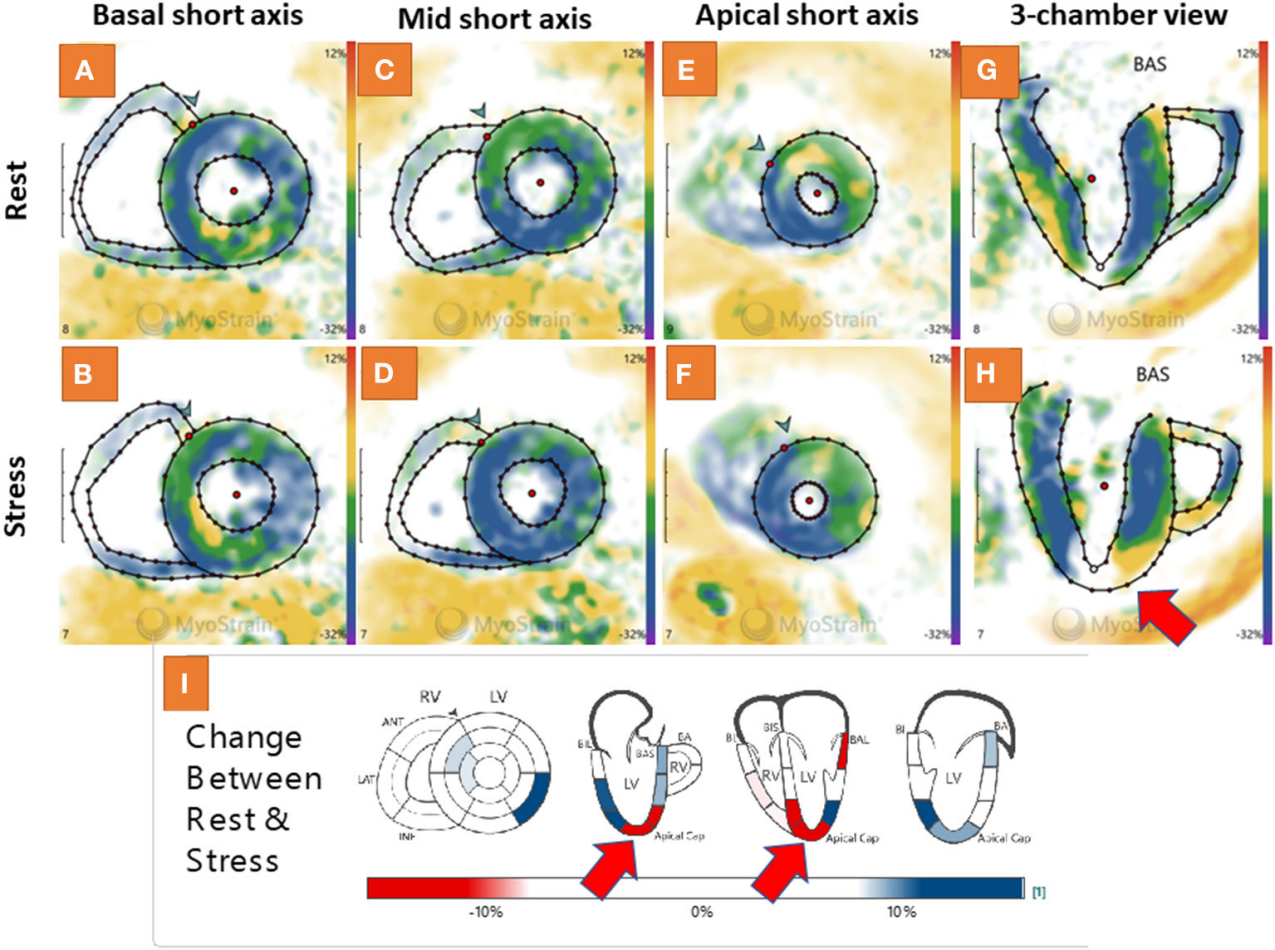
Adenosine stress cardiovascular magnetic resonance (CMR) showed normal findings in a 58-year-old female patient with suspected coronary artery disease (CAD) due to atypical angina (not shown). A normal strain response was also seen by fast-SENC in the short axis views **(A–F)**. However, abnormal strain response was detected in the corresponding 3 chamber view fast-SENC images **(G,H)** with a significant inducible strain decrease in the apical cap [red arrow in **(H)** and the corresponding bull's eye maps in **(I)**].

A 58-year-old female patient with arterial hypertension, hyperlipidemia and suspected CAD due to atypical angina underwent adenosine stress CMR, which did not show abnormal findings (not shown). Normal strain response was seen by fast-SENC in the short axis views (Figures 6A–F). However, abnormal strain response was detected in the corresponding 3 chamber view fast-SENC images (Figures 6G,H) with a significant inducible strain decrease in the apical cap (red arrow in Figure 6H and the corresponding bull's eye maps in Figure 6I). Due to the absence of a perfusion defect cardiac catheterization was deferred. However, after 1 year the patient underwent urgent revascularization by PCI and stent placement in the LAD.

## Discussion

In this analysis of 111 patients with complete perfusion, segmental strain analysis, and follow-up outcomes, we found that:

- Myocardial perfusion abnormalities during vasodilator administration were associated with clinical outcomes.
- Evaluation of segmental myocardial strain during baseline and vasodilator stress using fast-SENC was feasible with high reproducibility and within reasonable time-spent.
- An inducible absolute SAS decrease ≥6.5% during adenosine stress in one or more myocardial segments was associated with inducible perfusion abnormalities during first-pass perfusion imaging.
- Perfusion and strain abnormalities during vasodilator stress predicted future cardiac outcomes in patients with ischemic heart disease. Overall, perfusion defects showed numerically higher predictive values for future events, which was an expected finding since perfusion is a well-established marker for the prediction of future events (4, 7). However, segmental strain measures exhibited independent and incremental value for the prediction of outcomes.

### Previous Studies

We and others had previously described the incremental value of SENC both for the diagnostic classification and for the risk stratification of patients with suspected and known CAD (11, 12). Quantitative strain analysis enabled the identification of myocardial ischemia already during intermediate stages of inotropic stress, thus exhibiting enhanced sensitivity for the detection of CAD (12). In addition, the incremental value of SENC was demonstrated for the prediction of clinical outcomes during inotropic stress (11). Despite the presence of wide evidence for the diagnostic and prognostic value of inotropic strain in patients with ischemic heart disease, most of the clinical stress CMR studies are currently performed using vasodilator stress with adenosine or regadenoson. In this direction, the study of Romano et al. recently demonstrated the incremental value of longitudinal strain during vasodilator stress for the risk stratification of patients with CAD (25). Thus, using regadenoson perfusion stress CMR, GLS was measured at baseline and during stress in the 2-chamber long-axis cine view. GLS ≥ −19 during vasodilator stress was independently associated with worse cardiac outcomes, independent of clinical variables, perfusion abnormalities, and LGE data. This study reinforced the incremental value of strain assessment during vasodilator stress CMR. However, the strain was assessed by feature tracking imaging in this study, which was shown to have limitations, such as low reproducibility especially on a regional level and with less experienced operators (26–28). This, along with the fact that analysis was performed only in the 2-chamber long-axis view limits the interpretation of the obtained results (29). In this regard, fast-SENC provides an alternative to FTI and allows for a very quick, single heartbeat and comprehensive evaluation of regional and global myocardial strain with high reproducibility (18). In our study, SENC enabled the comprehensive assessment of myocardial strain with single heartbeat acquisitions with high reproducibility. Worsening myocardial strain during vasodilator stress CMR offered the precise detection of perfusion defects and was also associated with worse ischemic outcomes during follow-up. This may be a particular advantage in patients with contraindications for gadolinium-based contrast agents. Changes in SAS were much more sensitive than global strain (GLS, GCS, and GS) both for the detection of regional ischemia by perfusion analysis and for the prediction of outcomes. This is not striking since ischemia usually occurs regionally in one or two of three perfusion territories and not in all three simultaneously. In contrast to the study by Romano et al. (25) SCS and SAS were more strongly associated with perfusion abnormalities and predictive of outcomes compared with SLS. This may be attributed to methodological differences between FTI and SENC, the latter allowing for more comprehensive and reproducible measures of strain, especially on a regional level, which is decisive for the detection of ischemic heart disease (18, 26–31). Generally, CS is believed to be a more sensitive marker for subtle myocardial dysfunction in asymptomatic patients without any history of cardiovascular disease (32) and a more accurate marker of regional ischemic myocardial dysfunction, which is supported by the results of our study (33).

### Implementation of Our Findings Into the Current Clinical Context

Functional stress testing has been the non-invasive gold standard for the diagnostic work-up of patients with suspected and known CAD within the last decades, whereas its role in the diagnostic classification and risk stratification is widely accepted in patients with chronic coronary syndromes (CCS) based on current guidelines (2). Particularly CMR is currently acknowledged by clinicians as the clinical gold standard technique for the assessment of myocardial function, ischemia, and viability, if required all within a single examination, non-invasively and without radiation exposure for the patients. The amount of evidence for the applicability of dobutamine and vasodilator stress CMR for the detection of ischemia and the risk stratification of patients with CAD is large and has been highlighted in previous meta-analyses (6, 7). Based on the recent ISCHEMIA study, however, the role of coronary revascularization guided by the presence of myocardial ischemia has been questioned, since patients with stable coronary disease and moderate or severe ischemia, may not always profit in terms of outcomes from a primary revascularization strategy (34). However, patients with angina at baseline due to obstructive CAD improved in terms of limiting symptoms such as angina and exertional dyspnea from invasive strategies, which is an important cornerstone in the treatment of CAD (35). Furthermore, a recent meta-analysis demonstrated that patients with CCS randomized to elective revascularization vs. optimal medical treatment exhibited benefits in terms of cardiac survival, which improved with longer follow-up durations and were associated with fewer spontaneous myocardial infarctions (36). In addition, the MR-Inform study reinforced the role of vasodilator stress CMR in patients with stable angina and risk factors for CAD, being non-inferior to a primary invasive approach with X-Ray angiography and fractional flow reserve (FFR) measures with respect to clinical outcomes in patients with CCS (37).

From a pathophysiologic point of view, myocardial strain is more sensitive to disturbances of the myocardial metabolism or perfusion, which may be seen in the early stages of many cardiovascular disorders, including CAD and heart failure (38). In this regard, we recently demonstrated that even in patients at risk for heart failure and without coronary or structural heart disease, the impaired myocardial strain may be present as an early sign of subclinical cardiac dysfunction (19). In the present study, reduced strain response was seen in patients with myocardial ischemia during vasodilator stress, compared with those without perfusion abnormalities. Since hyperemic stress leads to redistribution of myocardial blood flow between the endocardium and epicardium (39), it is conceivable that endocardial strain is reduced due to impaired oxygen supply of the endocardium in regions with inducible ischemia, resulting in lower strain values in such regions. Thus, such individuals may have early damage of the sub-endocardial or mid-myocardial layers and therefore experience worse outcomes during follow-up. However, at present, it is not clear whether impaired strain during hyperemic stress requires a specific treatment, which merits further investigation in future studies.

### Limitations

Our cohort was relatively small and heterogeneous with a high percentage of patients with known CAD. This limits the extrapolation of our findings to lower risk cohorts. In addition, the follow-up duration was quite short and only 25 endpoints were recorded. Especially the small number of hard endpoints such as death and non-fatal myocardial infarction and the inclusion of only patients with CMR and complete follow-up data are limitations, that need to be accounted for when interpreting our results. In addition, patients who underwent elective revascularization within the first 90 days after abnormal stress perfusion by CMR were excluded from analysis, and no repeated CMR was performed after the revascularization procedures, which would have helped define the impact of residual ischemia on clinical outcomes. Furthermore, the cut-off value of SS decrease ≥6.5% during vasodilator stress was selected by *a priori* ROC analysis, as best indicative for the presence of myocardial ischemia by perfusion analysis, which may overestimate the association between perfusion defects and strain. However, the same cut-off was then independently applied for the prediction of cardiac events, exhibiting significant prognostic value. In addition, a certain overlap was observed for strain values in patients with and without perfusion abnormalities, so that average strain values may not be useful on an individual scale. However, by a selection of a cut-off value for the strain on a segmental level, clinically acceptable accuracy rates could be achieved for the estimation of perfusion abnormalities and clinical endpoints on a patient-by-patient level. Furthermore, we cannot evaluate the accuracy of strain or perfusion abnormalities for CAD detection by comparing these variables to invasive data in conjunction with FFR measures. However, most of our patients had negative stress test results, which helped defer invasive angiography, and this is clinically meaningful.

### Conclusions

Fast-SENC during vasodilator stress was non-inferior compared with standard visual perfusion analysis for the diagnosis of ischemia and the risk stratification of patients with ischemic heart disease. This may bear clinical implications since fast-SENC relies on purely quantitative analysis, which is reproducible and can be performed within reasonable time-spent. Thus, fast-SENC can obviate the need for contrast agent injections in this regard, in the interest of time and costs and potentially patient safety. Now prospective larger-scale trials are warranted to test the ability of fast-SENC to predict hard cardiac outcomes.

## Data Availability Statement

The raw data supporting the conclusions of this article will be made available by the authors, without undue reservation.

## Ethics Statement

The studies involving human participants were reviewed and approved by Ethics Committee of the General Medical Council Hamburg. The patients/participants provided their written informed consent to participate in this study.

## Author Contributions

GK and HS designed the study, performed the analysis, wrote and reviewed the manuscript, and provided important intellectual input. MM performed the acquisitions, reviewed the manuscript, and provided important intellectual input. SK, SE, AS, and SG reviewed the manuscript and provided important intellectual input. All authors contributed to the article and approved the submitted version.

## Conflict of Interest

HS, SK, and GK received research grants from Myocardial Solutions. SK owns stock options of Myocardial Solutions. The remaining authors declare that the research was conducted in the absence of any commercial or financial relationships that could be construed as a potential conflict of interest.

## Publisher's Note

All claims expressed in this article are solely those of the authors and do not necessarily represent those of their affiliated organizations, or those of the publisher, the editors and the reviewers. Any product that may be evaluated in this article, or claim that may be made by its manufacturer, is not guaranteed or endorsed by the publisher.

## Supplementary Material

Supplementary Figure 1 Corresponding mid short axis images in (i) a patient with normal perfusion during stress [**(A,B)**, note the asterisk, pointing to a dark rim artifact during resting perfusion], (ii) a patient with a subendocardial defect [**(C,D)**, red arrow in **(D)**, pointing to an inducible subendocardial perfusion defect of the septal wall] and (iii) a patient with a transmural perfusion defect [**(E,F)**, red arrow in **(F)**, pointing to an inducible transmural perfusion defect of the inferior-septal wall] during vasodilator stress are provided in this figure.

Supplementary Figure 2 On a segmental level, a slight absolute SLS decrease and a blunted SLS increase was observed in segments with subendocardial and transmural perfusion defects, respectively, vs. segments with normal perfusion during stress, which showed a slight SLS increase **(A)**. The same patterns were observed after exclusion of segments with LGE **(B)**.

Supplementary Table 1 Hemodynamic data during vasodilator stress.

## Abbreviations

AUC: area under the curve
BMI: body mass index
CABG: coronary artery bypass graft
CMR: cardiovascular magnetic resonance
CAD: coronary artery disease
CCS: chronic coronary syndromes
CS: circumferential strain
Fast-SENC: fast strain encoded sequence
GCS: global circumferential strain
GFR: glomerular filtration rate
GLS: global longitudinal strain
GS: global strain
LGE: late gadolinium enhancement
LS: longitudinal strain
LV: left ventricle
PCI: percutaneous coronary intervention
RV: right ventricle
SCS: segmental circumferential strain
SCS: segmental circumferential strain
SAS: segmental aggregate strain.
