# Supplementary figures and images for: Fast-Strain Encoded Cardiac Magnetic Resonance During Vasodilator Perfusion Stress Testing
Source: Front Cardiovasc Med. 2021 Nov 17;8:765961. doi: 10.3389/fcvm.2021.765961 (PMC8635645; doi:10.3389/fcvm.2021.765961)

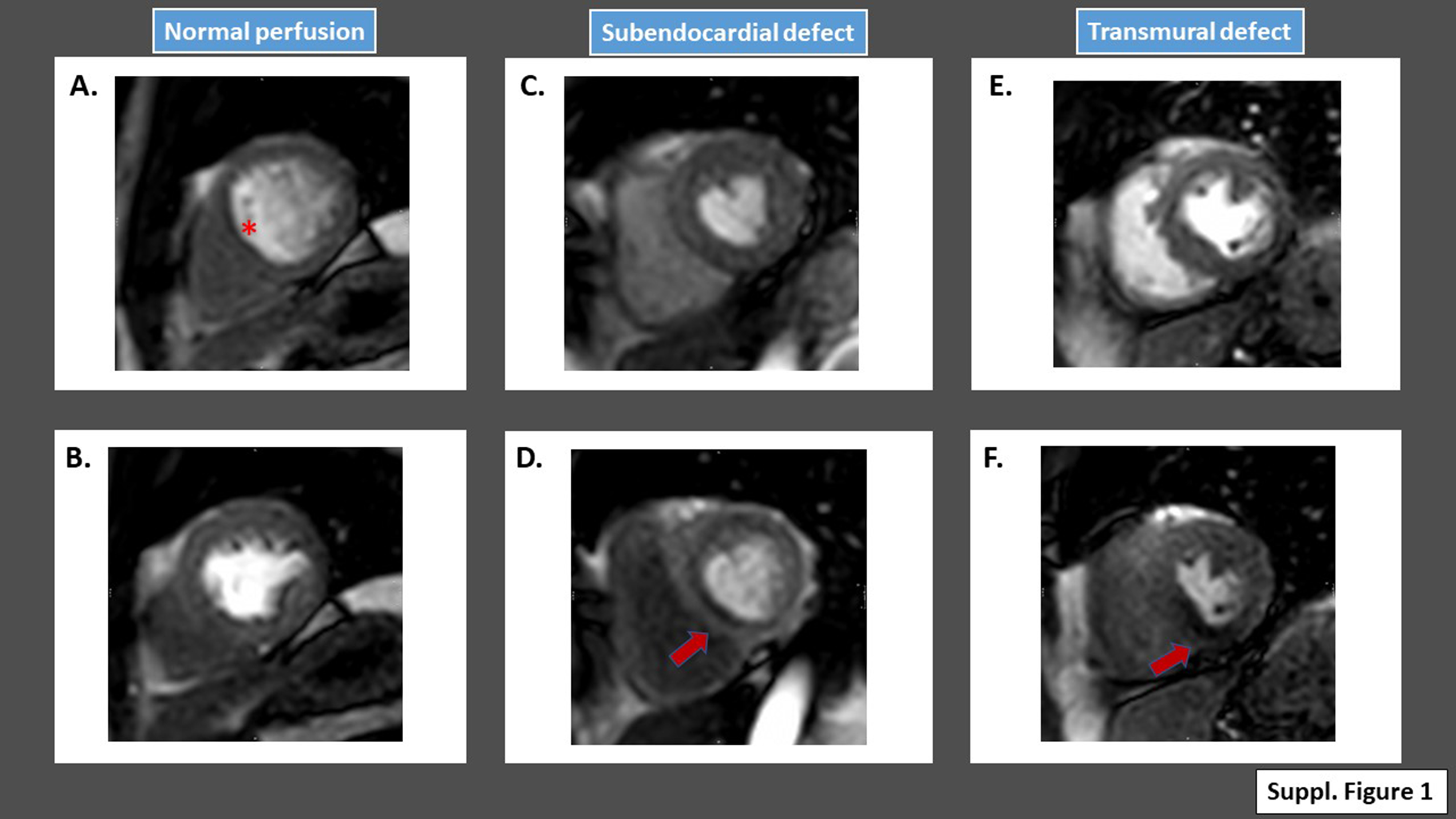

Supplement: Supplementary Figure 1 — Corresponding mid short axis images in (i) a patient with normal perfusion during stress [(A,B), note the asterisk, pointing to a dark rim artifact during resting perfusion], (ii) a patient with a subendocardial defect [(C,D), red arrow in (D), pointing to an inducible subendocardial perfusion defect of the septal wall] and (iii) a patient with a transmural perfusion defect [(E,F), red arrow in (F), pointing to an inducible transmural perfusion defect of the inferior-septal wall] during vasodilator stress are provided in this figure. [file Image_1.JPEG]

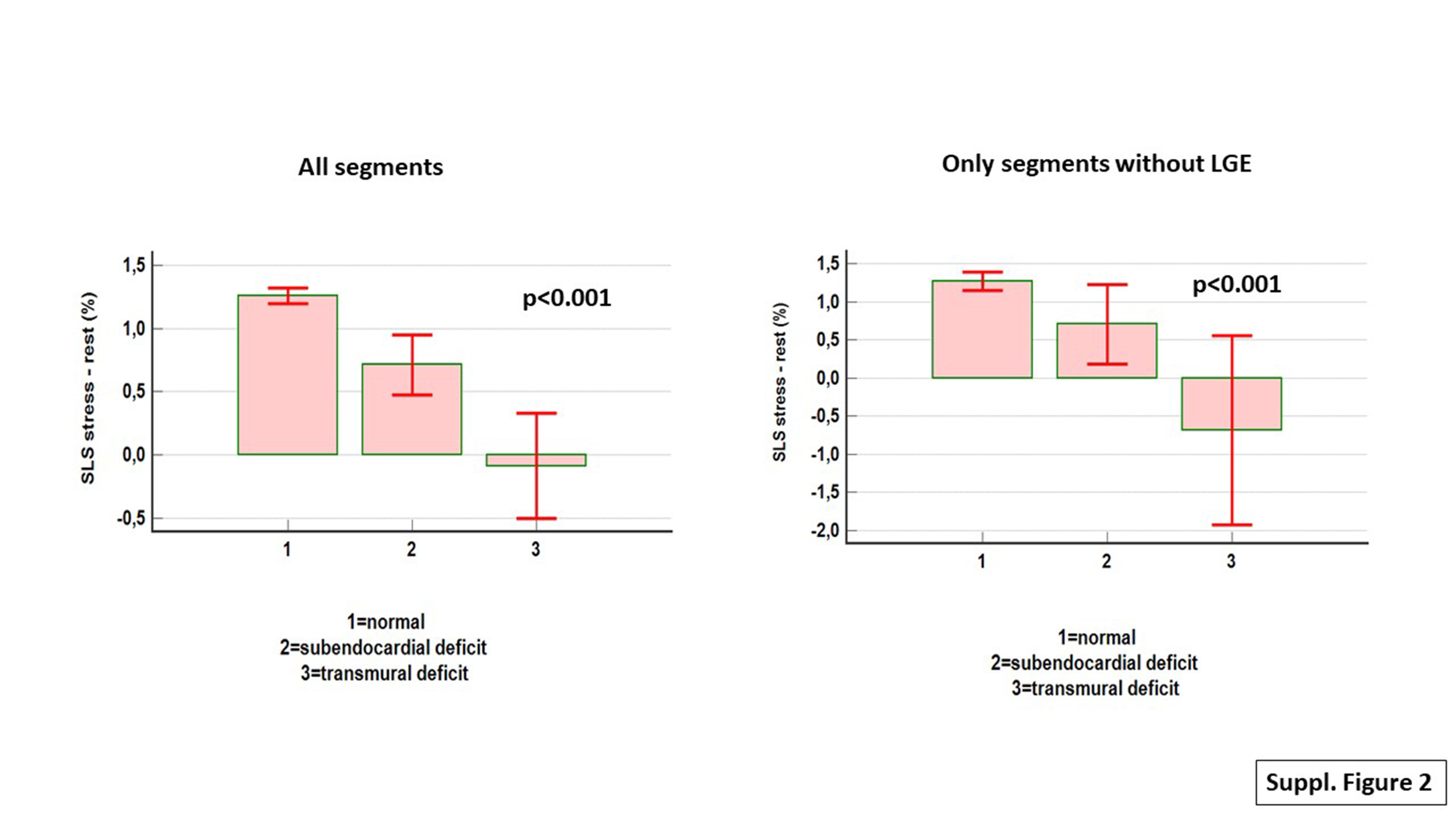

Supplement: Supplementary Figure 2 — On a segmental level, a slight absolute SLS decrease and a blunted SLS increase was observed in segments with subendocardial and transmural perfusion defects, respectively, vs. segments with normal perfusion during stress, which showed a slight SLS increase (A). The same patterns were observed after exclusion of segments with LGE (B). [file Image_2.JPEG]
