# Supplementary material for: Fast-Strain Encoded Cardiac Magnetic Resonance During Vasodilator Perfusion Stress Testing
Source: Front Cardiovasc Med. 2021 Nov 17;8:765961. doi: 10.3389/fcvm.2021.765961 (PMC8635645; doi:10.3389/fcvm.2021.765961)
Supplement: Supplementary Table 1 — Hemodynamic data during vasodilator stress. [file Table_1.docx]

**Supplementary Table 1.** Hemodynamic data during vasodilator stress.

| **Parameters** | **Heart rate (bpm)** | **Systolic blood pressure (mmHg)** | **Diastolic blood pressure (mmHg)** | **Rate pressure product (RPP in bmp*mmHg)** |
| --- | --- | --- | --- | --- |
| **Rest** | 66.4±10.2 | 138.8±19.7 | 80.2±11.5 | 9232±2048 |
| **Adenosine stress** | 88.9±16.7 | 134.3±20.7 | 77.1±12.3 | 11990±3192 |
| **p-values for rest versus stress** | p<0.001 | p=0.01 | p=0.002 | p<0.001 |
